# Fulvic acid application increases rice seedlings performance under low phosphorus stress

**DOI:** 10.1186/s12870-024-05435-4

**Published:** 2024-07-25

**Authors:** Xiaomeng Lv, Qingchao Li, Xuan Deng, Shitao Ding, Ruibo Sun, Shunquan Chen, Wenjing Yun, Changrong Dai, Bingbing Luo

**Affiliations:** 1 Anhui Province Key Lab of Farmland Ecological Conservation and Nutrient Utilization, Anhui Province Engineering and Technology Research Center of Intelligent Manufacture and Efficient Utilization of Green Phosphorus Fertilizer, College of Resources and Environment, Anhui Agricultural University, Hefei, 230036 P. R. China; 2 Jiangsu Key Laboratory for Bioresources of Saline Soils, Jiangsu Synthetic Innovation Center for Coastal Bio-agriculture, Jiangsu Provincial Key Laboratory of Coastal Wetland Bioresources and Environmental Protection, School of Wetlands, Yancheng Teachers University, Yancheng, 224007 China; 3 Bijie Academy of Agricultural Sciences, Bijie, 551700 China; 4 Shenzhen Institute of Molecular Crop Design, Shenzhen, 518107 China

**Keywords:** Fulvic acid, Rice seedlings, Low phosphorus stress, Physiological mechanism

## Abstract

**Background:**

Fulvic acid enhances plant growth and interacts synergistically with phosphate fertilizer to alleviate the agricultural production problem of low phosphorus fertilizer utilization efficiency. However, the underlying mechanism of its action remains poorly understood. In this study, we investigated the impact of fulvic acid application with varying concentrations (0, 40, 60, 80 and 120 mg/L) on rice performance in plants grown in a hydroponic system subjected to low phosphorus stress. The rice growth phenotypes, biomass, root morphology, phosphorus uptake, and the impact of fulvic acid on the rhizosphere environment of rice, were assessed.

**Results:**

The findings showed that adding appropriate concentrations of exogenous fulvic acid could promote the growth performance of rice under low phosphorus stress. Particularly at T1 (40 mg/L) and T2 (60 mg/L) over the control effectively increased rice biomass by 25.42% and 24.56%, respectively. Fulvic acid treatments stimulated root morphogenesis, up-regulated phosphate transporter genes, and facilitated phosphorus absorption and accumulation. Especially T1 (20.52%), T2 (18.10%) and T3 (20.48%) treatments significantly increased phosphorus uptake in rice, thereby alleviating low phosphorus stress. Additionally, fulvic acid elevated organic acids concentration in roots and up-regulated plasma membrane H^+^-ATPase genes, promoting organic acids secretion. This metabolic alteration can also alleviate low phosphorus stress in rice.

**Conclusions:**

The effect of exogenous fulvic acid on physiological indicators is concentration-dependent under low phosphorus stress, enhances rice performance and reduces reliance on phosphorus fertilizer. This provides new insights to shed light on the mechanism of alleviating low phosphorus stress in rice through fulvic acid application, an eco-friendly tool.

**Supplementary Information:**

The online version contains supplementary material available at 10.1186/s12870-024-05435-4.

## Introduction

Phosphorus (P) is an essential nutrient for plant growth and is a constituent of various plant metabolites and macromolecules. However, the availability of soluble inorganic phosphate (Pi) in soil that can be directly absorbable by plants typically occurs at low concentrations, limiting crop yield. Excessive P fertilizer is often applied to boost Pi levels and crop growth, but crop P fertilizer utilization efficiency (PFUE) remains below 30%, leading to wastage of phosphorus resources and environmental pollution [1, 2]. Enhancing PFUE is crucial, which is achieved by reducing P fixation and improving its bioavailability through combined soil conditioners and P fertilizer application.

Fulvic acid (FA), a component of humus substances, mainly comprises soluble organic compounds, and serves as a biological activator [3]. Its oxygenated carboxyl, carbonyl, phenolic hydroxyl, hydroxyl and quinone functional groups, can chelate or exchange ions [4, 5]. The unique composition of FA imparts biological and chemical activity [6]. For instance, it mitigates plant lead Pb toxicity by reducing Pb absorption and maintaining normal plant growth and development [7], and also enhances drought resistance [8]. Numerous studies have demonstrated the efficacy of FA in agriculture. FA application alleviates growth inhibition in continuously cropped potato seedlings [9], thereby functioning as a hormone-like substance and environmental response regulator to promote plant growth and development [10] Furthermore, FA reputedly improves maize photosynthesis and yield [11], while also effectively controlling apple tree canker, in a dose-dependent manner [12].

Humic substances (HSs), as a complex substance which contains humic acid (HA), fulvic acid (FA) and humin [13] increase plant nutrient absorption by improving root morphology [14, 15] and they affect the expression of nutrient transporter to make plats absorb these elements more effectively [16, 17]. Besides, HSs stimulate plant roots to secrete organic acids, facilitating interaction between plants and beneficial microorganisms [15, 18, 19]. It is worth further verifying whether FA, as one of the main components of HSs, has similar functions. Researchers have found that humic acid enhances P mobility by dissolving insoluble phosphates and promoting the formation of humic acid metal phosphate complexes [20]. FA can serve as a P fertilizer synergist, and directly regulates nutrient availability in soil and fertilizers, improving nutrient absorption, assimilation, root distribution, and growth stimulation [21, 22]. However, the effectiveness of FA is contingent upon soil conditions, fertilizers, and application methods. Because of its limited P utilization efficiency, rice which is a vital global food crop and dietary staple for many, often relies on phosphate fertilizer during its crucial seedling and tillering stages [23]. Therefore, exploring ways to increase P absorption in rice and soil P activation will lead to mitigating low P stress and enhance sustainable agricultural production. FA is an environmentally friendly P enhancer that has been studied mainly because of it can enhance PFUE and reduce crops’ excessive dependence on phosphorus fertilize.Our objectives are to identify the physiological mechanisms by which the appropriate concentrations FA alleviates low P stress in rice. We achieve this by growing rice seedlings in a hydroponic environment, subjecting them to various concentrations of FA in nutrient solutions with low P stress, and the impact of these variables on rice biomass, root morphology characteristics, P absorption, expression of P-transporter genes, and root organic acids secretion. These findings advance our understanding of the regulatory mechanism of FA in mitigating low phosphorus stress in rice and support the development of novel P fertilizers containing FA to enhance PFUE.

## Materials and methods

### Plant material, growth conditions and fulvic acid (FA) treatments

The experimental analysis and FA treatments were conducted using rice (*Oryza Sativa* L.ssp. Japonica, cv. Nipponbare). Rice seeds were sterilized with a 30% (v/v) sodium hypochlorite solution for 30 min followed by four to six rinses with ultrapure water. After germinating in the dark at 25 °C for 3 days [24], 7-day-old rice seedlings were transferred to a phytotron with a 14-hour light/10-hour dark photoperiod, and temperature cycling between 30 °C and 24 °C. The relative humidity was maintained at 70%. The nutrient solution was renewed every 4 days. Once uniform growth was achieved, seedlings were cultured with a normal 1/2 Kimura B nutrient solution until the fourth leaf appeared [24]. Subsequently, they received low-phosphate (Pi) treatment (10 µM Pi) at varied FA concentrations: 0 mg/L (control), 40 mg/L (T1), 60 mg/L (T2), 80 mg/L (T3), or 120 mg/L (T4). Besides, another treatment was to add 2-morpholinoethanesulfonic acid (MES), a pH buffer agent to nutrient solutions containing low-phosphate treatment and FA with varying concentrations (T1-T4). The cultivation conditions of rice seedlings remain unchanged. The FA used was purchased from Shanghai Acmec Biochemistry Co., Ltd (China). The chemical molecular formula of FA is C_14_H_12_O_8_ and its molecular weight is 308.24. The pH of 10% FA aqueous solution is about 2.5-3.0.

### Rice phenotype, biomass, and root morphology analysis

After 18 days of treatment at different FA concentrations, rice seedlings were harvested. The plant height and fresh weight of different parts were measured immediately post-harvesting. The plant samples were dried at 105℃ for 30 min, then further dried at 75℃ for 3 days to obtain dry weight biomass. The root-to-shoot ratio was calculated by dividing root by shoot fresh weights [24].

Root morphology was analyzed using fresh, intact root samples by a root scanner (Regent Instruments, Montreal, QC, Canada). The main indicators analyzed included root length, root surface area, root projection area, root average diameter, root volume, root tip number, branch number, and cross number. Root length represented the sum of primary roots, adventitious roots, and all lateral roots.

### pH and organic acids measurement

The initial pH of the treatment solution was adjusted to approximately 5.50. After 4 days of FA treatment at varying concentrations, the pH in the rice treatment solution was measured by pH meter (FE20, Mettler Toledo, Int. Inc.). A 100 ml sample from each treatment was freeze-dried and dissolved in 2 ml of ultrapure water [25]. Organic acids concentrations were determined via high-performance liquid chromatography (HPLC) (Agilent 1200) at a constant temperature of 30 °C. Standard solutions with concentrations of 10 mg/L, 50 mg/L, 100 mg/L, 200 mg/L, 500 mg/L, and 1000 mg/L were prepared, with standard solutions being varied forms of mixed organic acids (oxalic acid, formic acid, acetic acid, and malic acid). The internal standard curve displayed an R-squared value of 0.999. Analytical methods followed Wang et al. (2022) [26].

### Chlorophyll content measurement

Determination of chlorophyll content in rice leaves using the 90% ethanol extraction method [27]. The chlorophyll a (Ca) and chlorophyll b (Cb) concentrations were calculated with the formulas: Ca = 13.95*A665–6.8*A649 and Cb = 24.96*A649–7.32*A665, utilizing absorbances at 649 nm (A649) and 665 nm (A665). The total chlorophyll content (CT) was determined by summing the Ca and Cb concentrations: CT = Ca + Cb.

### Determination of nutrient elements in rice and calculation of phosphorus-related indexes

To assess nutrient element concentrations in rice plants, samples were initially dried at 80 ℃ for 3 days to obtain dry weights. Then, 0.1 g of each dried sample was ground and then digested with 5 mL of H_2_SO_4_ at 280℃. The resulting digested solution was diluted with ultra-pure water. Total nitrogen (N) concentration in the diluted digested solution was determined using an Auto Analyzer (SEAL, AA3, Germany) [28].

To determine concentrations of total phosphorus (P), total potassium (K), iron (Fe), and magnesium (Mg). 0.1 g dry samples were digested with 60% HNO_3_ at up to 180℃ by inductively coupled plasma-optical emission spectrometry (ICP-OES) (Thermo Fisher Scientific, Waltham, MA, USA) [24]. Element concentrations were calculated based on measured data and dry weights. Phosphorus accumulation in plant parts and plant phosphorus uptake were computed as follows: P accumulation (tissue) = P concentration (tissue) × dry weight (tissue); P uptake = P accumulation (root) + P accumulation (leaf sheath) + P accumulation (leaf blade).

### Gene expression analysis

RNA from rice plants treated with FA for 18 days in root, leaf sheath, and leaf blade tissues was extracted by using TRIzol reagent (Invitrogen, Thermos Fisher Scientific, Waltham, MA, USA) and then reverse transcribed into first-strand cDNA using a reverse transcription kit (TOYOBO, Osaka, Japan). SYBR^®^ qPCR Master Mix (Vazyme, Biotechnology, Nanjing, China) was used for quantitative real-time PCR(qRT-PCR). The internal reference gene was the rice housekeeping gene *OsActin1*. The qRT-PCR primers are presented in Supplementary Table S1 [24].

### Statistical analysis

All data were analyzed using IBM SPSS Statistics version 23 software and plotted using GraphPad 8.0 software. Significance was determined using Duncan’s one-way analysis of variance (ANOVA). Differences were denoted by different letters to signify significance. (*P* < 0.05).

## Results

### Effects of fulvic acid on rice growth and development under low phosphorus stress

**Fig. 1:**
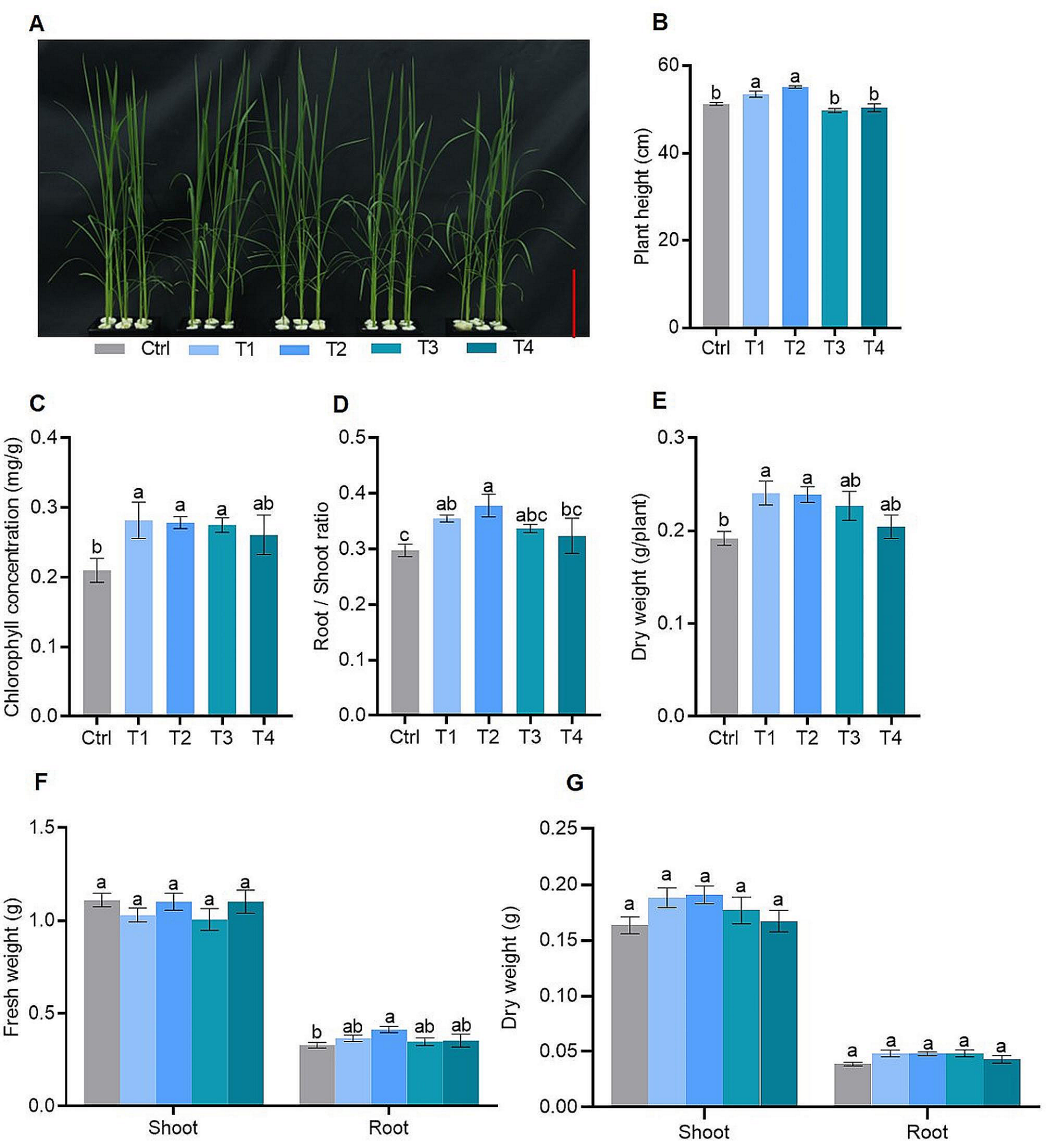
Effect of different fulvic acid concentrations on rice growth under low P (10 µM Pi in nutrient solution) stress: (**A**) the phenotypes, (**B**) plant height, (**C**) chlorophyll concentration, (**D**) root /shoot ratio, (**E**) dry weight of per plant (**F**) fresh weight and (**G**) dry weight of shoot and root. Ctrl (Control), T1, T2, T3 and T4 represent fulvic acid concentrations of 0, 40, 60, 80, 120 mg/L in the nutrient solution, respectively. Data are shown as means ± SE (*n* = 5). Different letters indicate significant differences between treatments (*P* < 0.05, one-way analysis of variance, Duncan’s test). The scale bar in (**A**) equals to 10 cm

Different concentrations of FA treatments significantly affected rice growth phenotypes (Fig. 1A). The plant height significantly increased in T1 (40 mg/L FA) and T2 (60 mg/L FA) compared with the control (0 mg/L FA), while there was no significant difference in plant height between the control, T3 (80 mg/L FA), and T4 (120 mg/L FA) (Fig. 1B). The chlorophyll concentration in leaves also increased by 34.31% (T1), 32.75% (T2), 31.02% (T3), and 24.37% (T4) compared with the control (Fig. 1C).The root/shoot ratio peaked in T2 treatment (Fig. 1D) The dry weight of per plant indicated 25.42%, 24.56%, 18.22%, and 6.58% increased for T1, T2, T3 and T4 compared with the control (Fig. 1E). The root fresh weight initially increased and later decreased with increasing FA concentrations, while rice shoot fresh weight remained consistent (Fig. 1F).Additionally, statistical analysis of dry weight in different tissues revealed that there was no significant difference in dry weight of shoot and root under different treatments, but the FA treatments showed an increasing trend compared to the control (Fig. 1G). The shoot dry weight of T1, T2, T3 and T4 increased by 15.11%, 16.68%, 8.18% and 2.32%, and the root dry weight of T1, T2, T3 and T4 increased by 24.45%, 24.19%, 24.70% and 11.02%, compared with the control (Fig. 1G). The most favorable results for plant biomass occurred in T2 treatment (60 mg/L).

### Fulvic acid affects the root morphology of rice under low phosphorus stress

**Fig. 2:**
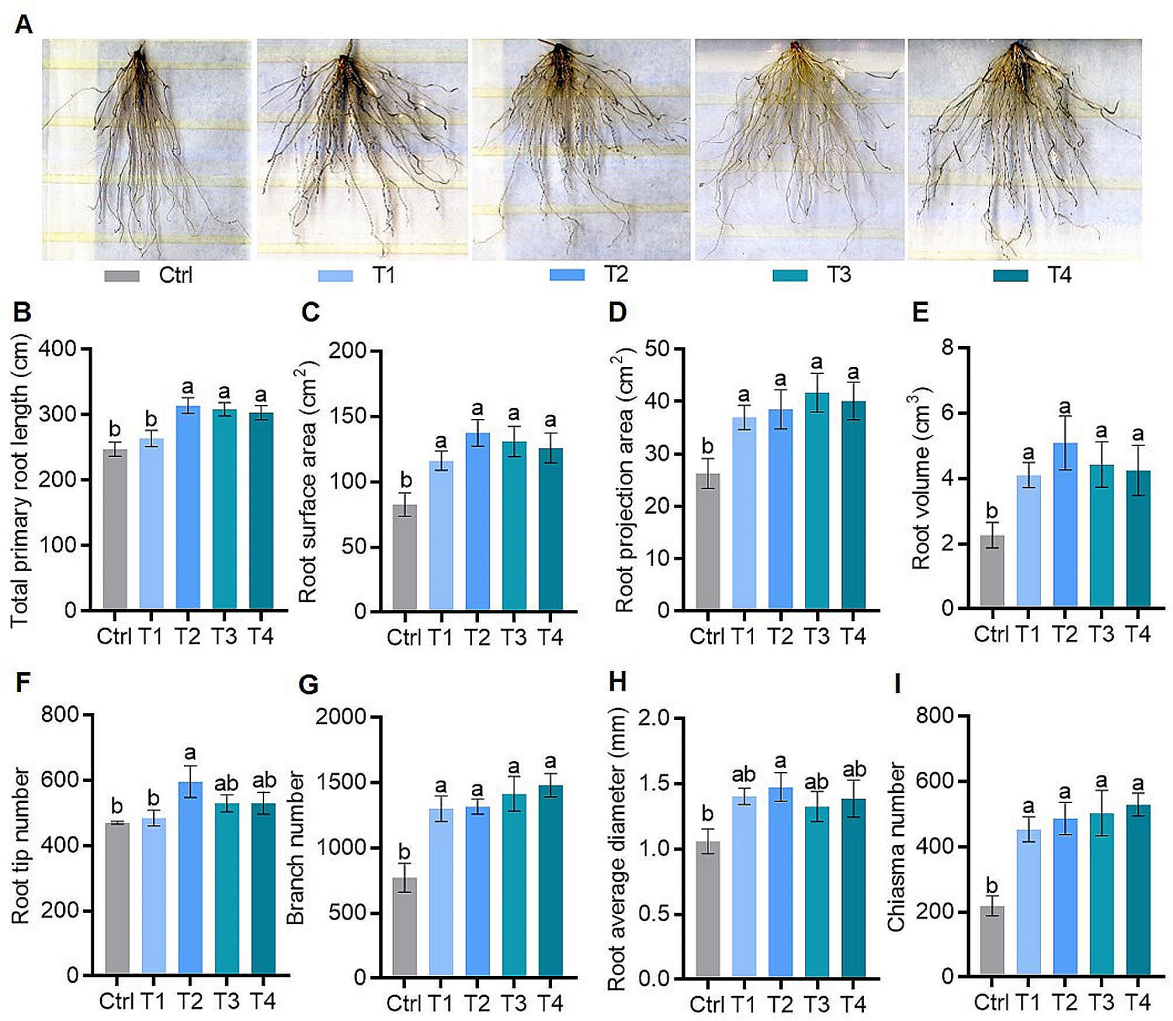
Effects of different-concentration fulvic acid on rice root morphology under low P (10 µM Pi in nutrient solution) stress: (**A**) root scanning photographs, (**B**) total primary root length, (**C**) root surface area, (**D**) root projection area, (**E**) root volume, (**F**) root tip number, (**G**) branch number, (**H**) root average diameter and (**I**) chiasma number. Ctrl (Control), T1, T2, T3 and T4 represent fulvic acid concentrations of 0, 40, 60, 80, 120 mg/L in the nutrient solution, respectively. Data are shown as means ± SE (*n* = 5). Different letters indicate significant differences between treatments (*P* < 0.05, one-way analysis of variance, Duncan’s test)

To assess the effects of varying FA concentrations under low P conditions on rice root morphology by using the root scanner. The resulting images showed root angles in FA treatments were expanded compared with the control (Fig. 2A). This suggested that the external application of FA could significantly increase root length, root surface area, root projection area, root volume, root average diameter, root tip number, branch number, and chiasma number of rice with the control. However, there were no significant differences among treatments (Fig. 2). Notably, traits such as total primary root length, root surface area, projection area, volume, branch number, and chiasma number improved with increasing FA concentrations (Fig. 2B-E, G and I). Therefore, FA application promoted rice root growth, potentially improving P acquisition under low P-stress environment.

### The impact of fulvic acid on rice nutrient absorption under low phosphorus stress

**Fig. 3:**
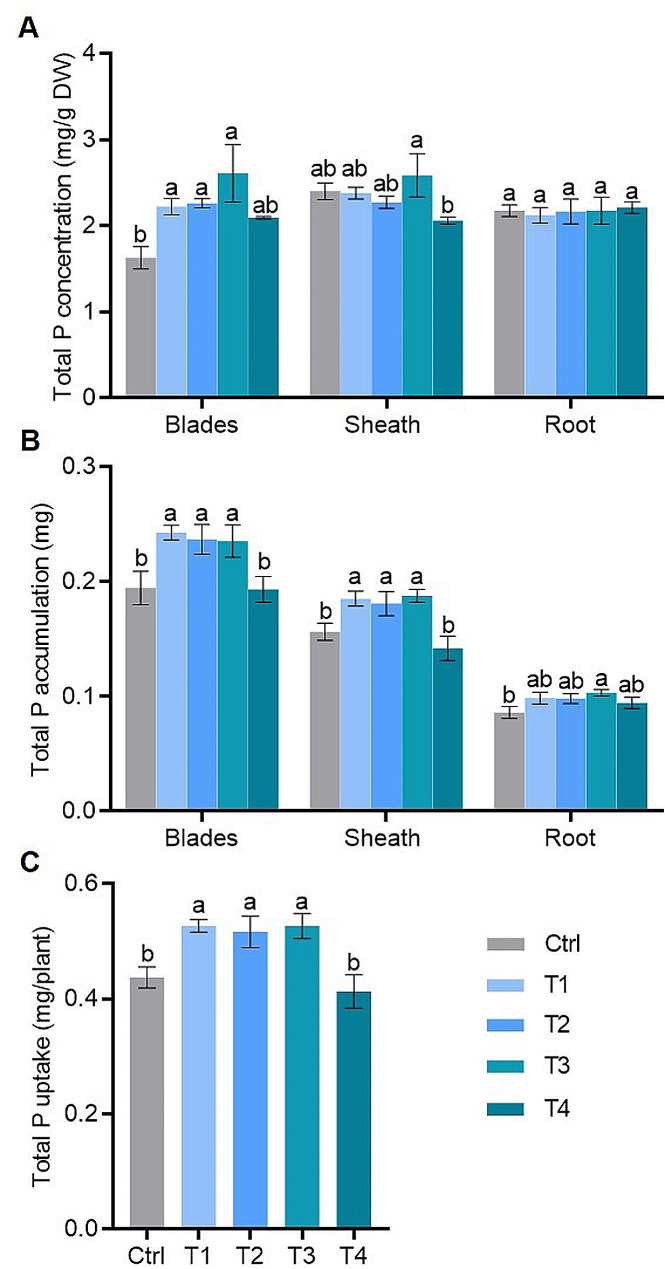
Fulvic acid promotes P concentration and accumulation of rice under low P (10 µM Pi in nutrient solution) stress: (**A**) the total P concentration in different parts of rice, (**B**) the total P accumulation in different parts of rice and (**C**) total P uptake in a whole seedling. Ctrl (Control), T1, T2, T3 and T4 represent fulvic acid concentrations of 0, 40, 60, 80, 120 mg/L in the nutrient solution, respectively. Data are shown as means ± SE (*n* = 5). Different letters indicate significant differences between treatments (*P* < 0.05, one-way analysis of variance, Duncan’s test)

The effect of FA application on nutrient concentrations was shown in Fig. 3 and S1. Figure 3A displayed P concentration in various tissues of the rice plant. Expecting T4, the P concentration of leaf blades was significantly higher in all FA treatments than in the control. For leaf sheathes and roots, there were no significant differences in P concentration between FA treatments and control. Leaf blades and sheaths had significantly higher P accumulation in all FA treatments compared to the control and T4 FA treatment (Fig. 3B). Additionally, the roots in the T3 FA treatment saw a significant 19.8% increase in P accumulation compared with the control, P accumulation trended upwards in other FA treatments (Fig. 3B). Furthermore, the P uptake by rice plants revealed significant increases in rice treated with T1, T2, and T3 by 20.52%, 18.10%, and 20.48%, respectively, while no significant difference was observed in the T4 FA treatment (Fig. 3C). These findings indicated that an appropriate FA concentration enhanced P absorption and accumulation in rice, alleviating the adverse effects of low P stress.

Concurrently, concentrations of other nutrient elements (N, K, Mg, Fe) in various parts of the rice plant were measured for each FA treatment (Figure S1). There were no significant differences in total N and K concentrations compared with control (Figure S1 A and B). Mg levels were 11.53% lower in T4 sheaths but 16.41% higher in T1 roots compared with control (Figure S1 C). Fe concentrations in rice blades, sheaths, and roots were all higher in FA treatments compared with control (Figure S1 D). These results suggested that an appropriate exogenous FA concentration could enhance both P absorption and Fe concentration in rice under low P stress.

### Effects of fulvic acid on the expression of phosphate transporter genes in rice under low phosphorus stress

**Fig. 4:**
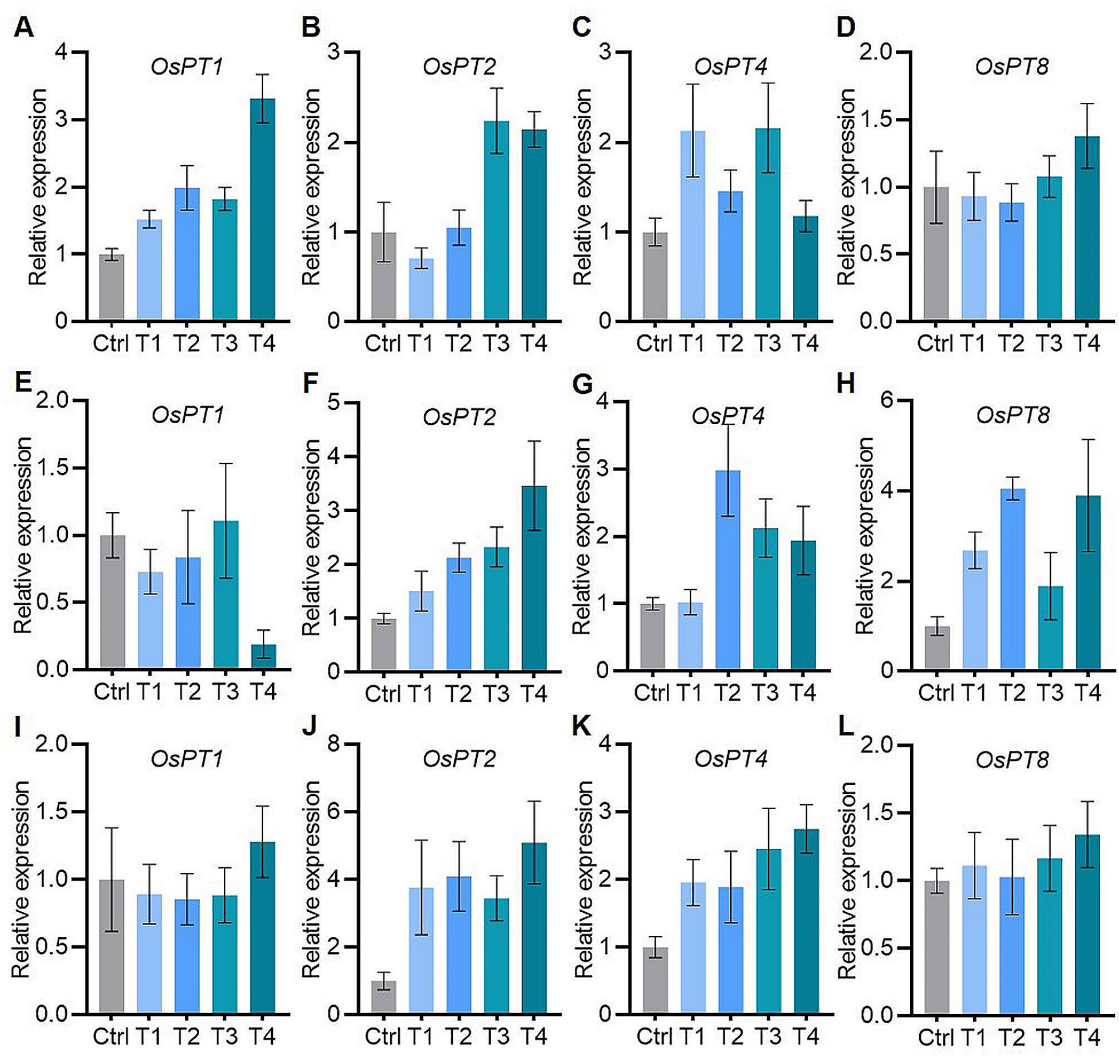
Effects of fulvic acid on the expression of phosphate transporter genes in rice. (**A**)-(**D**), (**E**)-(**H**) and (**I**)-(**J**) were the relative expression levels of *OsPT1*, *OsPT2*, *OsPT4* and *OsPT8* in blades, sheath and root in different FA treatments under low P (10 µM Pi in nutrient solution) stress, respectively. Ctrl (Control), T1, T2, T3 and T4 represent fulvic acid concentrations of 0, 40, 60, 80, 120 mg/L in the nutrient solution, respectively. The expression value of each gene in the control treatment was set as 1. *OsActin1* was used as the reference gene. Data are shown as means ± SE (*n* = 5 plants)

P is primarily absorbed as Pi from the rhizosphere and subsequently transported with in various tissues. Crucial phosphate transporters (*PTs*) in rice, such as *OsPT1*, *OsPT2*, *OsPT4*, and *OsPT8*, play a pivotal role in phosphate acquisition and transport. To elucidate the potential mechanisms behind increased P concentrations in plants under FA treatment, we analyzed the transcriptional expression patterns of *PTs* in rice under various FA treatments in low P conditions (Fig. 4).

Expression levels of *OsPT1* and *OsPT4* in leaf blades were consistently higher in all FA treatments compared with control, *OsPT2* and *OsPT8* were up-regulated just in T3 and T4 (Fig. 4A-D). Similarly, the expression of *OsPT2* and *OsPT8* in sheath tissue increased in all FA treatments compared with the control, while *OsPT4* was more highly expressed in T2, T3, and T4. Notably, Relative expression of *OsPT1* in sheath tissue decreased in T4, otherwise for this gene, there were no significant differences in other FA treatments compared with the control (Fig. 4E-H). In root tissue, *OsPT2* and *OsPT4* were significantly up-regulated in all FA treatments, while the expression of *OsPT1* and *OsPT8* was unaffected by FA treatment (Fig. 4I-L). These findings indicated that enhanced P concentrations and accumulation in rice tissues following FA treatments under low P stress were associated with up-regulation of phosphate-transporter genes.

### Effects of fulvic acid on organic acids secretion and gene expression related to rice root response to low phosphorus stress

**Fig. 5:**
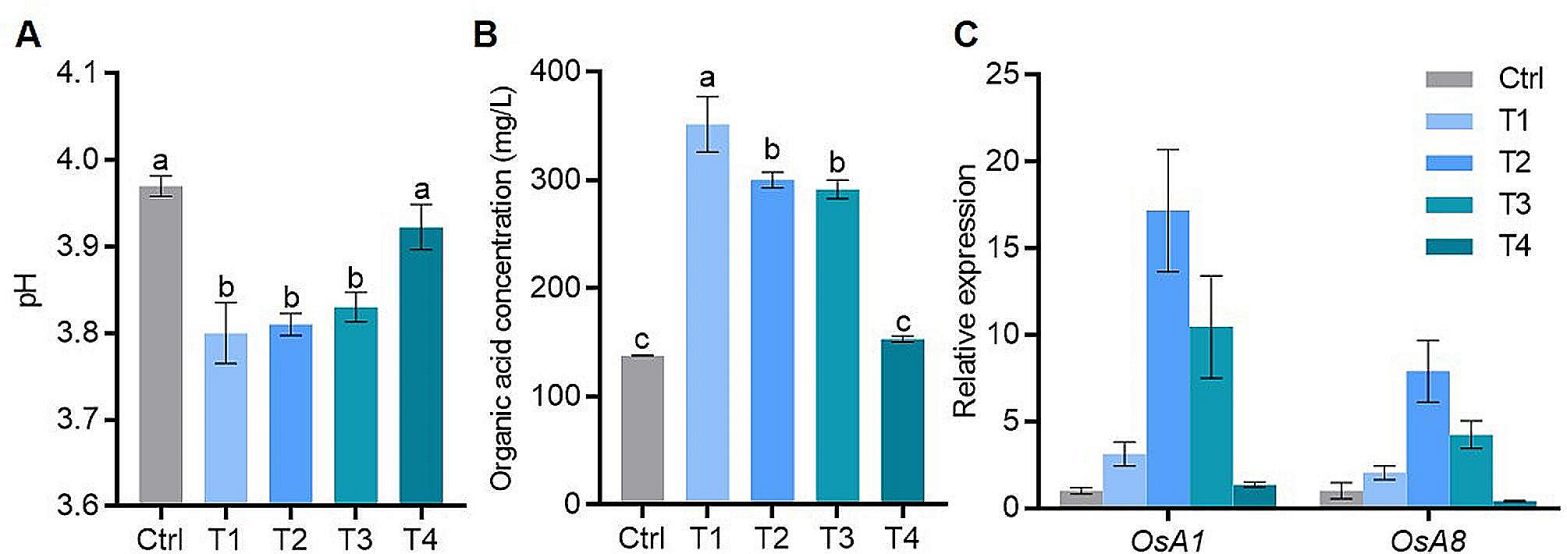
Effect of fulvic acid on secretion of organic acids and the expression of H^+^-ATPase genes in rice root under low P (10 µM Pi in nutrient solution) stress. (**A**) and (**B**) were pH values and concentrations of root-secreted organic acids in nutrient solutions containing different concentrations of fulvic acid after cultivating rice seedlings for 4 days. (**C**) was the relative expression of two H^+^-ATPase genes, *OsA1* and *OsA8*, in rice roots under different fulvic acid treatments. Ctrl (Control), T1, T2, T3 and T4 represent fulvic acid concentrations of 0, 40, 60, 80, 120 mg/L in the nutrient solution, respectively. The expression value of each gene in the control treatment was set as 1. *OsActin1* was used as the reference gene. Data are shown as means ± SE (*n* = 5)

Expecting T4, the nutrient solution pH was significantly lower in FA treatments compared with the control (Fig. 5A). Concurrent with pH changes, assessment of organic acids excretion by rice roots under FA treatments showed that total organic acids concentration also significantly increased in FA treatments (T1-T3) compared with the control, but there was no significant difference between T4 and the control (Fig. 5B). Changes in pH values and organic acids are often accompanied by alterations in the expression of plasma membrane H^+^-ATPase genes [29]. *OsA1* and *OsA8* are primary plasma membrane H^+^-ATPase genes in rice responsible for H^+^ efflux and nutrient absorption [30, 31]. Therefore, the relative expression of *OsA1* and *OsA8* in roots in FA treatments was presented in Fig. 5C. Excepting T4, the expression of *OsA1* and *OsA8* were upregulated in FA treatments compared with the control (Fig. 5C). Therefore, an appropriate FA concentration addition could induce up-regulation of H^+^ efflux-related gene expression, promote organic acids secretion in roots, lead to external root growth environment acidification. This acidification enhances phosphorus activation and absorption, and aids low P-stress tolerance.

Furthermore, we introduced 2-morpholinoethanesulfonic acid (MES), a pH buffer agent, to the nutrient solutions in various treatments to study the impact of FA on rice growth under low P conditions. Interestingly, the phenotypes, plant height, and dry weight did not differ from control values following the addition of MES in any FA treatment (Figure S2 A-C). Additionally, contrary to findings in Fig. 3, the P concentration, P accumulation, and total P uptake in sheaths and roots in FA treatments were unchanged little or even significantly decreased in blades compared with the control (Figure S2D-F). These results suggested that the addition a pH buffer agent inhibited the positive impacts of FA on rice growth and P uptake under low P stress.

## Discussion

The function of FA in promoting crop development and growth has been reported [32, 33]. FA with high physiological activity stimulates plant growth, nutrient absorption, photosynthetic efficiency, and crop resistance [34, 35]. Adding humic acid-like substances to soil can enhance crop phosphorus utilization to varying degrees [36, 37]. In this study, the application of FA with varying concentrations was related to different growth parameters of greenhouse rice seedlings under low phosphorus conditions. Adding a small amount of humic acids significantly promoted root growth and increased crop yield [38]. As expected, our study indicated that adding an appropriate concentration of FA can alleviate rice growth and development under low phosphorus stress.

Phosphorus deficiency inhibits biomacromolecule synthesis and energy metabolism, reduces photosynthetic rate, and hampers carbon assimilation, impeding plant growth [39]. Applying an optimal FA concentration significantly enhances rice biomass and chlorophyll levels, mitigating low P stress. Additionally, exogenous FA application also boosts rice’s P absorption capacity under low P stress, in a concentration-dependent manner. This is attributed to FA’s numerous active functional groups, which mimic hormones and yield pleiotropic effects [10]. Changes in root morphology affect essential nutrient absorption by roots [40]. FA application stimulates lateral root and root hair proliferation, enhances the differentiation rate of root cells, and alters root configuration [32]. Besides, humic acid-like substances have auxin-like functions [41]. Auxin has the effect of promoting crop root growth [42]. These root morphological changes increase root-nutrient contact, and in doing so, improve Pi absorption.

At the molecular level, phosphate transporter genes (*PTs*) are important in Pi acquisition and transport. It has been reported that tomato roots treated with humic acids induced overexpression of *PTs* [16]. In this study, FA application facilitates Pi absorption and transport in rice by up-regulating related *PTs*. OsPT2 and OsPT4 manage Pi absorption under exogenous FA addition at low P stress. Appropriate FA concentration not only induced *OsPT2*, *OsPT4*, and *OsPT8* up-regulation in sheaths that participate in Pi transport but also *OsPT1*, *OsPT2*, and *OsPT4* expression in leaves involved in Pi transport and distribution. Therefore, *OsPT1*, *OsPT2*, *OsPT4*, and *OsPT8* are all involved in Pi uptake in rice roots [24, 43]. In particular, *OsPT1* and *OsPT8* exhibited relatively high expression in the root and shoot [44]. OsPT2 transported Pi from the root to shoot [43], and OsPT4 affected Pi mobilization in rice [45].

Phosphorus deficiency affects plant growth and induces the release of organic acids by plant roots. By lowering rhizosphere pH, it consequently increases P availability in the soil [46]. Exogenous FA at an appropriate concentration, further promotes organic acid secretion by roots. However, the addition of a pH buffer to stabilize growth medium pH eliminated low-P resistance in rice under FA treatments, underscoring the direct influence of rhizosphere pH on the resistance of rice to external low P stress.

Organic acid excretion depends on plasma membrane H^+^-ATPase activity, which expels H^+^ ions through ATP hydrolysis to maintain cellular charge balance [47]. Knowing that HSs activated the plasma membrane H^+^-ATPase [48] and up-regulating the plasma membrane H^+^-ATPase gene in Arabidopsis enhances proton-pump activity to promote P absorption [49]. Our study observed up-regulation of plasma membrane H^+^-ATPase genes *OsA1* and *OsA8* at appropriate FA concentrations. Knocking out *OsA8* significantly reduces the ability of rice to absorb and transport P [31, 50], establishing a strong correlation between plasma membrane H^+^-ATPase and P absorption.

## Conclusion

**Fig. 6:**
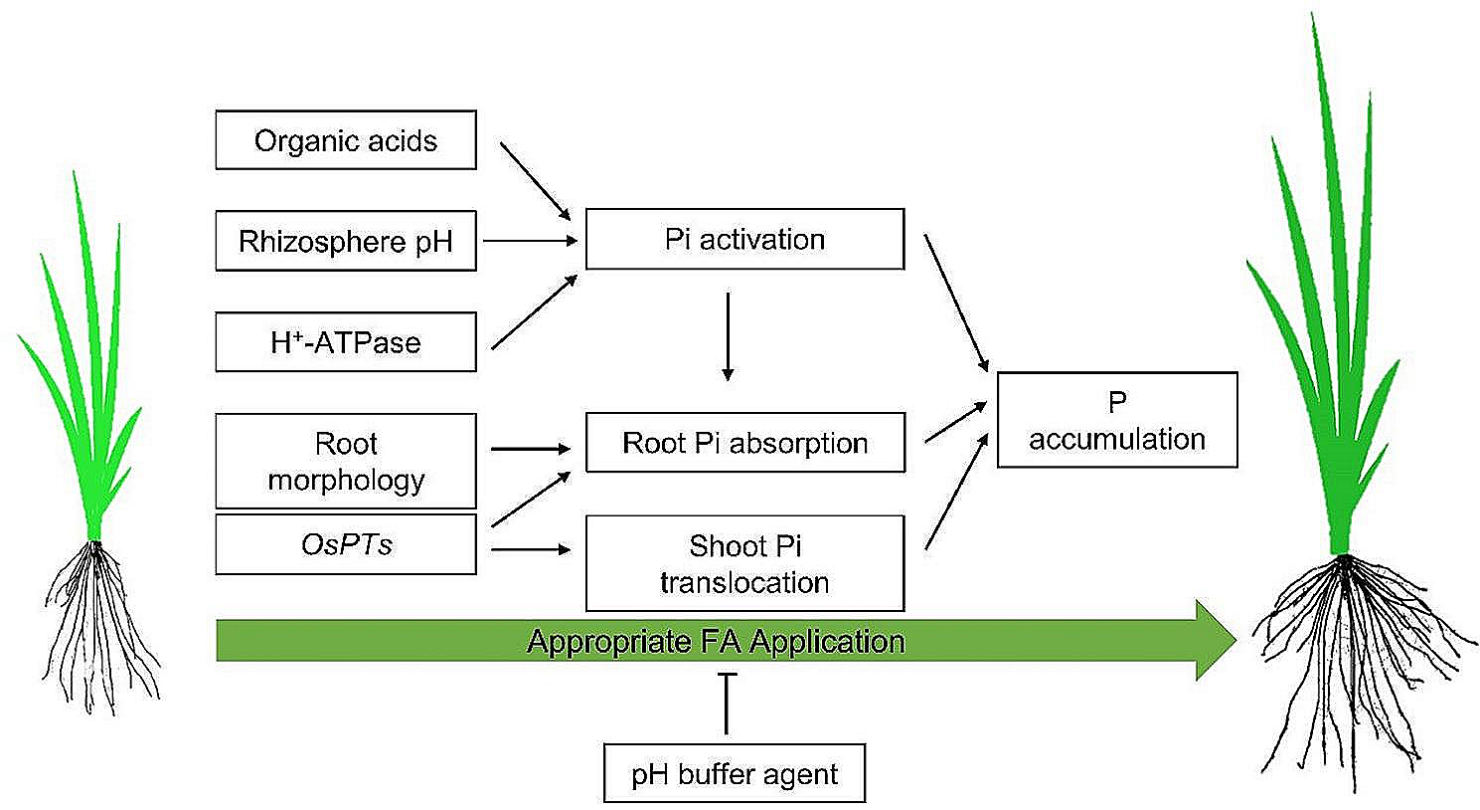
Working model of fulvic acid (FA) promoting P accumulation of rice under low P stress. Appropriate FA application influences the root-secreted organic acids, rhizosphere pH, expression of H+-ATPase genes, root morphology and expression of *OsPTs*. Positive changes in root-secreted organic acids, rhizosphere pH and expression of H^+^-ATPase genes can promote Pi activation, which can cooperate with changes in root morphology and *OsPTs* abundance to increase Pi absorption. Furthermore, OsPTs can help to promote shoot Pi translocation. The synergistic effect of the above factors promotes phosphorus accumulation. Nevertheless, the facilitation of P accumulation caused by appropriate FA application can be inhibited by pH buffer agent

We reported the positive effects of FA on rice plants subjected to low P stress, possibly through the mechanism illustrated in Fig. 6. FA addition at an appropriate concentration sequentially alters root morphology, up-regulates root plasma membrane H^+^-ATPase gene expression, promotes organic acids secretion, and reduces pH in the rice growth environment, which may facilitate decomposition of chelated P compounds and increases phosphorus and iron elements absorption by the roots. FA application at an appropriate concentration elevates PT expression and improves P transport from roots to shoots. Further research is required to identify precisely the regulatory mechanism of how FA mitigates against low P stress in rice.

### Electronic supplementary material

Below is the link to the electronic supplementary material.

Supplementary Material 1

## Data Availability

The data supporting the present study’s findings are available from the corresponding author upon reasonable request.
