# Supplementary material for: Fulvic acid application increases rice seedlings performance under low phosphorus stress
Source: BMC Plant Biol. 2024 Jul 25;24:703. doi: 10.1186/s12870-024-05435-4 (PMC11271057; doi:10.1186/s12870-024-05435-4)
Supplement: Supplementary file 1 — Supplementary Material 1 [file 12870_2024_5435_MOESM1_ESM.docx]

**Table S1.** The primers used for real-time qRT-PCR

| Gene name | Primer name | Sequence |
| --- | --- | --- |
| *OsActin1* | OsActin1-F | CAACACCCCTGCTATGTACG |
|  | OsActin1-R | CATCACCAGAGTCCAACACAA |
| *OsPT1* | OsPT1-F | CGCTTCCGTACGAGTGGTAGT |
|  | OsPT1-R | GGTTCTTTCAAATCCAGGGAAA |
| *OsPT2* | OsPT2-F | GACGAGACCGCCCAAGAAG |
|  | OsPT2-R | TTTTCAGTCACTCACGTCGAGAC |
| *OsPT4* | OsPT4-F | TATTGCGGCTTAGATTGCATTAG |
|  | OsPT4-R | TCCAAATCAAATGGGCACTAAG |
| *OsPT8* | OsPT8-F | AGAAGGCAAAAGAAATGTGTGTTAAAT |
|  | OsPT8-R | AAAATGTATTCGTGCCAAATTGCT |
| *OsA1* | OsA1-F | GTGTTTGGGTTTATGCTGCT |
|  | OsA1-R | GTATCCACCCAGCACAACTC |
| *OsA8* | OsA8-F | TCAACCAAATGGCTGAAGAG |
|  | OsA8-R | CCACAGATTCCACCTTTCCT |


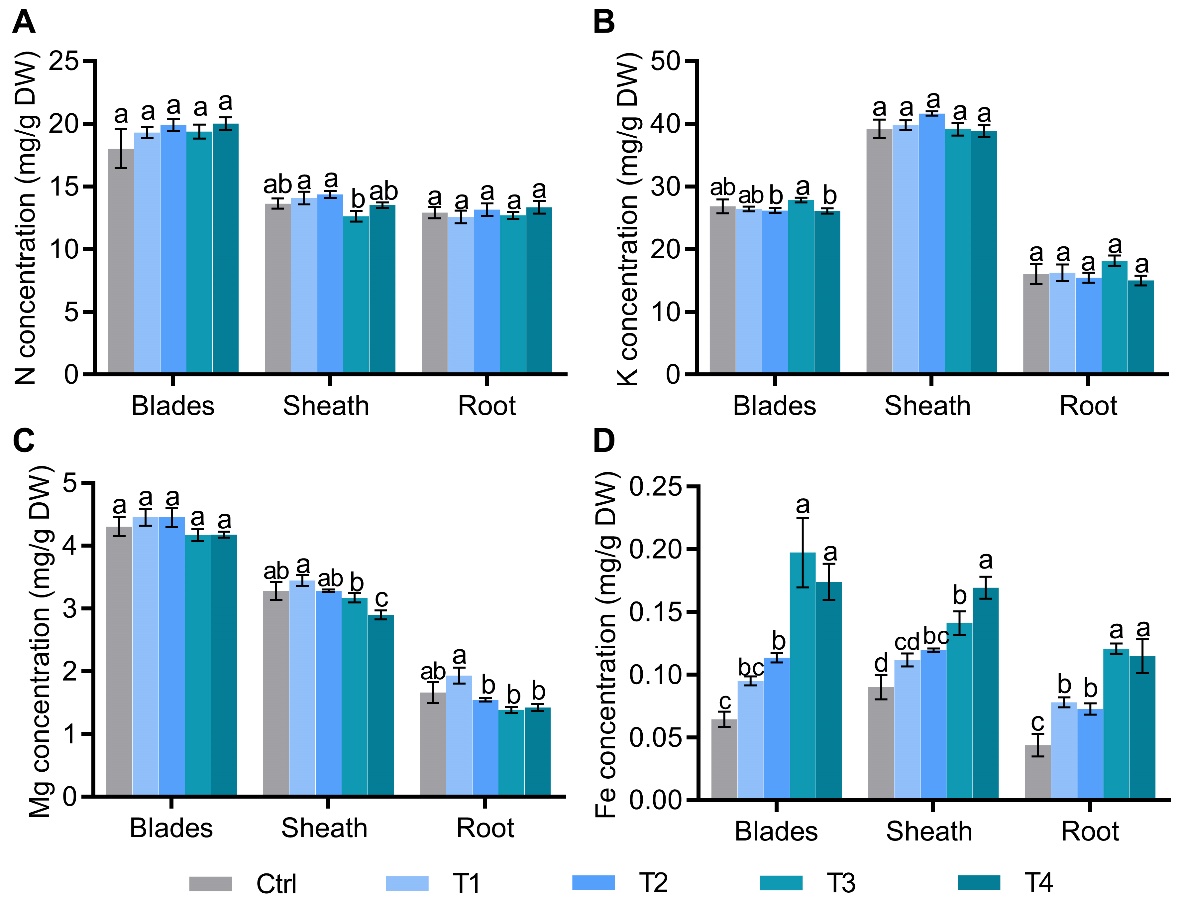


**Figure S1.** Effects of fulvic acid on elements of rice in different FA treatments under low P stress. (A)-(D) are the concentrations of N, K, Mg and Fe of different parts in different FA treatments under low P stress, respectively. Ctrl (Control), T1, T2, T3 and T4 represent FA concentrations of 0, 40, 60, 80, 120 mg/L in the nutrient solution, respectively. Data are shown as means ± SE (*n* = 5). Different letters indicate significant differences between treatments (*P* < 0.05, one-way analysis of variance, Duncan’s test)


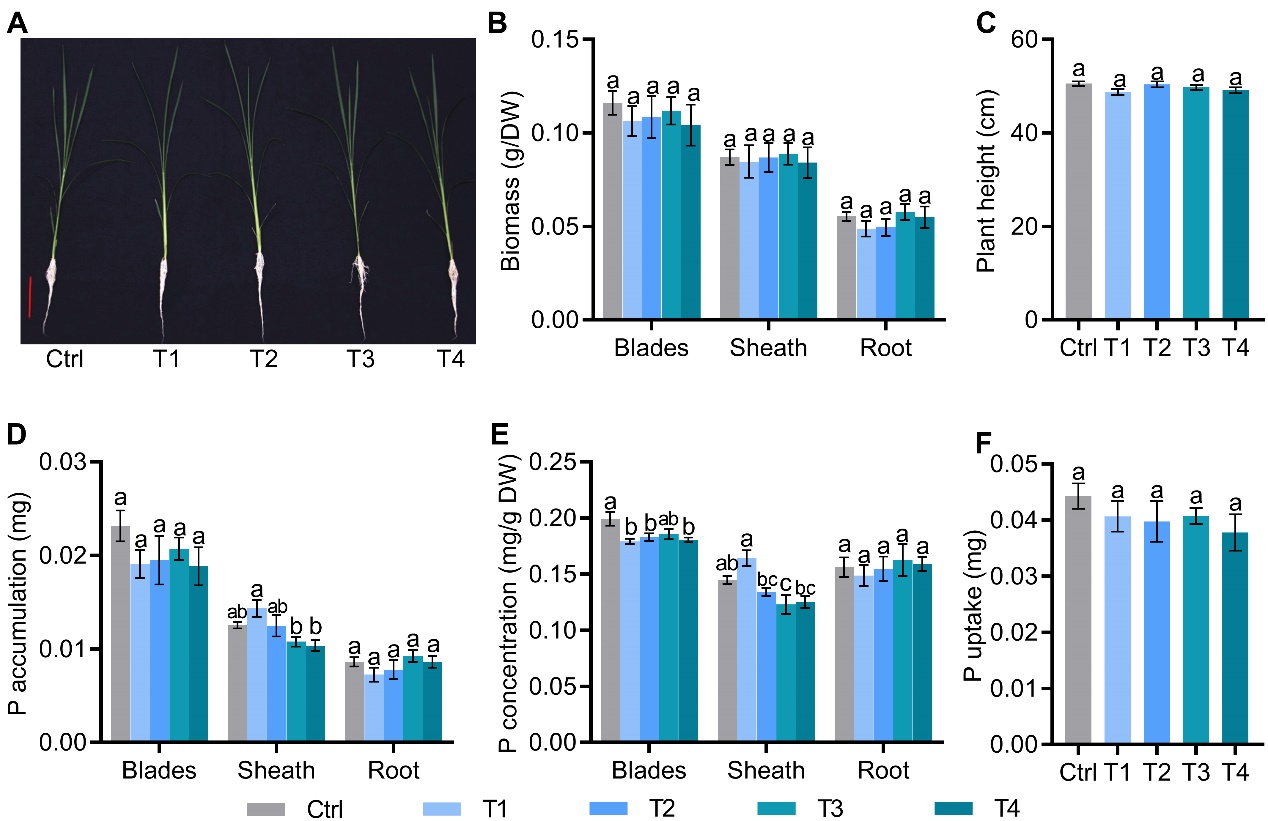


**Fig S2.** Adding pH buffer inhibits the effect of fulvic acid on rice growth and P absorption. MES (2-morpholinoethanesulfonic acid) was added to the nutrient solution of different FA treatments as the pH buffer agent. (A)-(F) were the phenotypes, biomass, plant height, the P concentration in different parts of rice, the P accumulation in different parts of rice and total P uptake in a whole seedling, respectively. Ctrl (Control), T1, T2, T3 and T4 represent FA concentrations of 0, 40, 60, 80, 120 mg/L in the nutrient solution, respectively. Data are shown as means ± SE (*n* = 5). Different letters indicate significant differences between treatments (*P* < 0.05, one-way analysis of variance, Duncan’s test). The scale bar in (A) equals to 10 cm
